# Nucleophilic Addition versus Migratory Insertion Pathways in the Gold‐Catalyzed Heck Reaction: A Computational Study

**DOI:** 10.1002/chem.202501645

**Published:** 2025-07-07

**Authors:** Peter H. M. Budzelaar, Luca Rocchigiani, Manfred Bochmann

**Affiliations:** ^1^ Department of Chemistry University of Naples Federico II Via Cintia Naples I‐80126 Italy; ^2^ Department of Chemistry, Biology and Biotechnology University of Perugia Via Elce di Sotto 8 Perugia I‐06134 Italy; ^3^ School of Chemistry University of East Anglia Norwich Research Park Norwich NR4 7TJ UK

**Keywords:** catalysis, chelate ligands, DFT, gold, mechanisms

## Abstract

The initial reaction steps in the formation of Heck‐type arylated alkenes catalyzed by P^N chelated Au(III) complexes have been studied computationally. Two mechanistic alternatives have been explored: (1) alkene coordination and insertion into a gold‐carbon bond, and (2) nucleophilic attack on a gold(III) alkene adduct. The common starting point, the [(P^N)AuPh(alkene)]^2+^ dication (alkene = C_2_H_4_, H_2_C═CHEt, or H_2_C═CEt_2_), shows unequal bonding to the olefinic carbons of the 1‐alkenes (P^N = 1,2‐C_6_H_4_NMe_2_(PR_2_); R = H, Me, 1‐adamantyl). This polarization increases with steric hindrance and with the inclusion of an OTf^‐^ anion in the model. While the reaction pathways are strongly governed by the *trans*‐influence of the ligand, the effects of steric hindrance in the ligand and alkene are remarkably small. In all cases the nucleophilic attack pathway is energetically favored. However, changing the ligand from a P^N to a P^P chelate, with a strongly electron‐donating ‐PMe_2_ donor *trans* to Ph, sufficiently destabilizes the Au─Ph bond to make alkene insertion competitive. Alkene 1,2‐insertion regiochemistry is always preferred, unlike Pd‐catalyzed Heck reaction that requires a 2,1‐insertion. Based on these results, an alkene insertion pathway en route to Heck‐type olefins can therefore be ruled out.

## Graphical abstract

The “gold‐catalyzed Heck reaction” could in principle proceed either via nucleophilic attack by triflate, or by migratory alkene insertion. This DFT study shows that nucleophilic attack is strongly preferred but ligand dependent.

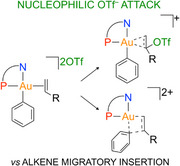

## 1 Introduction

Gold catalysts^[^ ^1^, ^2^ ^]^ have become widely established in synthesis protocols, as demonstrated, for example, by several monographs,^[^ ^3^, ^4^, ^5^ ^]^ and themed issues of major review journals.^[^ ^6^, ^7^, ^8^, ^9^, ^10^ ^]^ Catalytic cycles based on transition metal complexes typically consist of a number of key reaction steps, such as substrate coordination, intra‐ or intermolecular nucleophilic attack, insertions of unsaturated substrates into M─X bonds (X = H, C, O, etc.), and termination reactions such as reductive elimination and β‐H elimination.^[^ ^11^, ^12^, ^13^ ^]^ While several of these have been outlined in stoichiometric and catalytic reactions of gold complexes,^[^ ^14^ ^]^ it has also become evident that gold displays conspicuous reactivity differences from other transition metal catalysts.

In particular, much research has focused on the stoichiometric and catalytic chemistry of gold(III) compounds and gold(I, III) redox systems.^[^ ^15^, ^16^, ^17^ ^]^ Gold(III) naturally invites comparison with palladium(II) since both ions have a d^8^ electron configuration. However, although these two elements display very comparable structural chemistry, there are numerous examples where gold follows pathways that circumvent conventional mechanistic assumptions. These studies have also highlighted two other aspects of gold(III) chemistry: the dominating influence of the electron‐donor strength of the ligand *trans* to the reactive site (*trans*‐influence),^[^ ^18^ ^]^ and the high Lewis acidity of the Au(III) ion, particularly in cationic complexes.^[^ ^15^, ^19^ ^]^ The latter renders Au(III) alkene and alkyne complexes extremely electrophilic.^[^ ^20^, ^21^ ^]^ Standard reactions like migratory insertions into M─C bonds and β‐H elimination reactions of metal alkyls, which are so familiar in palladium catalysis,^[^ ^11^, ^12^, ^13^ ^]^ are the exception rather than the rule in gold chemistry.

The following examples may serve to illustrate this point. Isolable alkene complexes of Au(III) compounds^[^ ^20^, ^22^ ^]^ have proved highly susceptible to attack by nucleophiles such as water or alcohols, and while the overall outcome of such reactions may look like the result of migratory insertion into Au─O bonds, detailed studies by Tilset et al. have shown that they are the product of external attack by nucleophiles, including weak nucleophiles like trifluoroacetate.^[^ ^22^ ^]^ Furthermore, in recent years a range of gold(III) hydrides supported by bidentate and tridentate ligands has been synthesized,^[^ ^23^, ^24^, ^25^, ^26^, ^27^ ^]^ and the reactivity of these long‐elusive species can now be studied. It could be shown that insertions into Au(III)─H bonds can follow various pathways, depending on the *trans*‐influence of the ligand and the redox properties of the substrate. For example, certain isolable gold(I) and gold(III) hydrides with strong *trans*‐influence donors give insertion products with easily reducible activated alkynes, such as acetylene dimethylcarboxylate (DMAD), but fail to react with dialkyl or diaryl acetylenes.^[^ ^26^, ^28^, ^29^, ^30^ ^]^ In these cases it was proposed that one‐electron reduction of DMAD may generate the corresponding alkyne anion radical in the solvent cage, followed by radical dimerization. Alkyne radical anions are known to adopt a *trans* structure, which explains the stereochemistry.^[^ ^26^ ^]^ By contrast, gold(III) hydrides stabilized by C^N^C pincer ligands, which place a weak N‐donor *trans* to H, do not react with DMAD; they do however form *trans*‐insertion products with a wide variety of non‐activated alkynes. Such pincer complexes do not possess the vacant coordination site required for substrate coordination, and it could be shown that instead a bimolecular pathway involving Au(II) radicals is followed.^[^ ^31^ ^]^ A similar bimolecular Au(II) radical pathway also operates for the insertion of alkenes^[^ ^32^ ^]^ and of isocyanides^[^ ^33^ ^]^ into Au(III)─H bonds. None of the reactions of isolable or spectroscopically detectable gold(III) hydrides with alkenes or alkynes follows a migratory insertion pathway.^[^ ^34^ ^]^

The migratory insertion of nonactivated alkenes into Au─C single bonds has been unequivocally established only in a few cases, enabled by Bourissou's strongly electron‐donating phosphinonaphthyl P^C^(‐)^ chelate ligand, which weakens the reacting Au─C bonds by the strong *trans*‐influence of the dialkylphosphine donor. Migratory insertion into a gold‐methyl bond was observed for norbornene but not for ethylene or styrene,^[^ ^35^, ^36^, ^37^ ^]^ while analogous (P^C)Au‐aryl compounds insert ethylene.^[^ ^38^ ^]^ These complexes also show reversible β‐H elimination.^[^ ^39^ ^]^ DFT calculations showed that the activation barriers for ethylene insertion and for β‐H elimination are low, <10 kcal/mol.^[^ ^38^, ^39^ ^]^ However, replacing the P^C^(‐)^ ligand by the weaker *trans*‐effect analogue N^C^(‐)^ blocks both alkene insertion and β‐H elimination.^[^ ^40^ ^]^

**Scheme 1:**
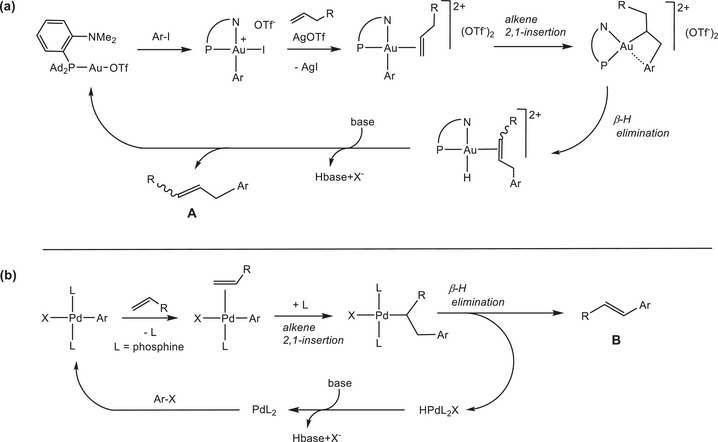
a) Hypothetical formation of benzyl alkenes **A** via a migratory insertion mechanism.^[^ ^41^ ^]^ b) Mechanistic outline of the Mizoroki‐Heck reaction, leading to styrene derivatives **B**.

Recently, Patil and co‐workers described the formation of Heck‐type arylated olefins from aryl iodides and unactivated 1‐alkenes such as 1‐hexene, catalyzed by (MeDalPhos)AuCl activated with silver salts (MeDalPhos = 1,2‐C_6_H_4_NMe_2_(PAd_2_); Ad = 1‐adamantyl). The proposed mechanism involved as a key step the migratory 2,1‐insertion of the alkene into the Au‐aryl bond, followed by β‐H elimination (Scheme 1a).^[^ ^41^, ^42^ ^]^ The products were non‐conjugated benzyl alkenes of type **A**. These alkene products differ, therefore, from the conjugated arylated olefins **B** that are formed in the well‐known palladium‐catalyzed Mizoroki‐Heck reaction.^[^ ^43^ ^]^ Notably, palladium catalysis involves a 2,1‐alkene insertion into a Pd‐aryl bond to give a branched metal alkyl, rather than the generally preferred 1,2‐insertion regiochemistry (Scheme 1b).^[^ ^44^ ^]^

If correct, the gold‐catalyzed Heck reaction would indeed be a remarkable and surprising extension of gold(III) chemistry. Unfortunately, the proposal relied solely on a DFT model that did not explore alternative scenarios,^[^ ^41^ ^]^ while adequate supporting experimental evidence for the insertion mechanism was lacking. However, there were several reasons to question the proposed mechanism: (i) MeDalPhos is a dialkylaryl phosphine with an insufficiently strong *trans*‐influence to facilitate alkene insertion into Au─C bonds. (ii) The β‐H elimination mechanism necessarily postulates the formation of the hypothetical [(P^N)AuH]^2+^ dication. Dicationic gold hydrides are unknown; they would be extremely electrophilic and in a medium containing bases like pyridine would be expected to be deprotonated immediately. Indeed, the authors’ own calculations^[^ ^41^ ^]^ showed that the rearrangement [(P^N)AuH(alkene)]^2+^ → [(P‐NH^(+)^Au]^+^ + alkene is strongly exergonic (ΔG = ‐25.4 kcal/mol). The N‐protonated cation [(P‐NH^(+)^Au]^+^ is of course perfectly stable but catalytically inactive; its formation would immediately shut down the catalysis. (iii) Problematic regiochemistry: The formation of Heck‐type arylated olefins requires 2,1‐alkene insertion, to give sterically demanding *branched* metal alkyls. On the other hand, calculations show that 1,2‐insertions into Au─C bonds are energetically preferred, leading to *linear* gold alkyls; hence this pathway would not lead to the observed 1‐aryl alkene products of type **A**.

**Scheme 2:**
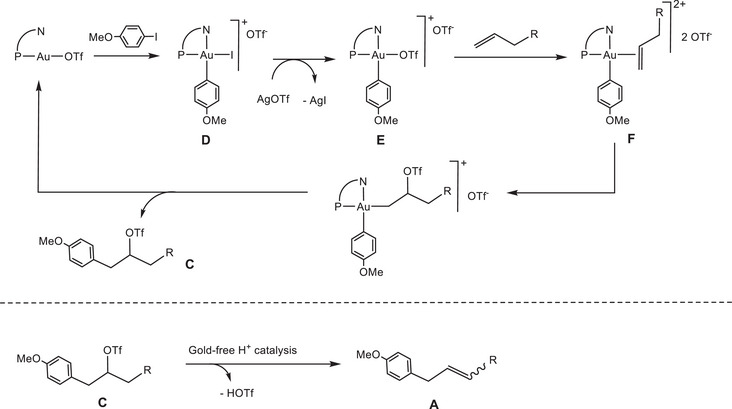
Two‐step formation of benzyl alkenes **A** via a gold‐catalyzed heteroarylation mechanism, followed by gold‐free H^+^‐catalyzed HOTf elimination.^[^ ^45^ ^]^

We showed recently that this so‐called “gold‐catalyzed Heck reaction” proceeds, in fact, by a completely different, insertion‐free mechanism (Scheme 2), which consists of two steps: (1) a gold‐catalyzed alkene heteroarylation to give the triflate ester **C**, where the triflate anion acts as a nucleophile toward a strongly electrophilic [LAu^(III)^(Ar)(alkene)]^2+^ dication, followed (2) by a proton‐catalyzed carbocationic reaction of the primary organic product to form the observed arylated olefins **A**.^[^ ^45^ ^]^ This second step proceeds in the complete absence of gold catalysts with identical results. Our study was supported by DFT calculations of the gold‐catalyzed sequence, using ethylene as a simplified substrate to minimize conformational issues.

Nevertheless, Patil's proposed Heck‐type scheme consisted of perfectly reasonable elementary steps, as also confirmed by DFT studies. Thus, it seemed not inconceivable that for slightly different choices of ligands, a Heck‐type mechanism involving migratory olefin insertion could become the preferred one (likely with quite a different regioselectivity). With this possibility in mind, we decided to explore the chemical landscape leading to Heck‐type arylated alkenes, and in particular the role(s) of DalPhos (P^N) ligands in this context. The diverse chemistry supported by the MeDalPhos ligand has been documented and has in part been attributed to the hemilability of the dimethylaminophenyl ligand arm.^[^ ^46^, ^47^, ^48^, ^49^, ^50^, ^51^ ^]^ This reactivity includes the insertion of CO (but not alkenes) into Au‐biphenylyl bonds, but only in the absence of nucleophiles.^[^ ^50^ ^]^ Thus, we have examined computationally various parameters that could be expected to lead to differences in reaction outcomes and explore here the influence of ligand steric hindrance, donor type, and the effect of alkene substituents, by comparing ethylene results with those of 1‐butene as well as 2‐ethyl‐1‐butene as a branched analogue. We also wished to explore under which conditions migratory 1‐alkene insertion into the Au─Ph bond might be preferred over nucleophilic attack by a weak nucleophile, such as the triflate anion, and which regiochemistry is preferred. As previously reported,^[^ ^46^, ^52^, ^53^ ^]^ key access to the cycle is provided by the oxidative addition of iodoarenes to (N─P)AuOTf to give the square‐planar intermediate **D** (Scheme 2).^[^ ^54^ ^]^

## 2 Results and Discussion

All calculations were done using Gaussian‐16 (rev C01)^[^ ^55^ ^]^ in combination with an external optimizer.^[^ ^56^ ^]^ Structures were fully optimized, without symmetry restrictions, using the dispersion‐aware MN15 functional,^[^ ^57^ ^]^ the cc‐pVDZ(pp)^[^ ^58^ ^]^ basis set^[^ ^59^ ^]^ on all atoms, and the SMD continuum solvation model for dichloromethane.^[^ ^60^ ^]^ All stationary points were verified (through vibrational analysis) to be minima (no negative Hessian eigenvalues) or transition states (exactly one negative eigenvalue corresponding to the correct reaction). Further details, tables of total and relative energies, and xyz structure archives are available as Supporting Information.

The influence of ligand structure was explored for the series:


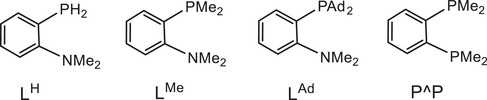


with increasing steric hindrance for P^N chelates.

In our model we replace the experimentally used *p*‐C_6_H_4_(OMe) by a Ph group; comparison with one test series (using the L^Me^ model ligand) showed that this makes virtually no difference. In all cases the preferred structures of the gold complexes are those with N placed *trans* to the electron‐donating phenyl ligand. Low‐lying reaction paths where the ‐NMe_2_ moiety leaves the coordination sphere of Au(III) could not be identified.

The P^P ligand offers a significantly different electronic environment since it places the strong *trans*‐influence donor PMe_2_ *trans* to the phenyl ligand, weakening the Au─Ph bond. In fact, the difference between P^P and the various P^N systems studied here is so large that one cannot expect straightforward translation of reaction mechanisms. Nevertheless, we feel that including such a variation can help to understand the factors determining regiochemistry.

Olefin substrates with increasing degrees of steric hindrance were considered: ethylene, 1‐butene as the most similar but simpler substitute for the 1‐hexene substrate used in the original studies,^[^ ^41^, ^42^ ^]^ and 2‐ethyl‐1‐butene.

**Scheme 3:**
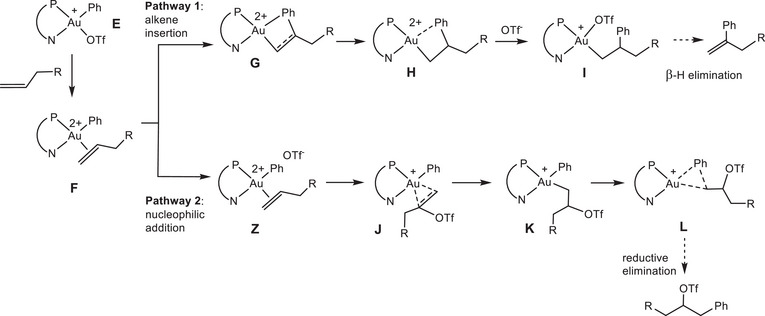
Computed reaction paths for the migratory alkene insertion (pathway 1) and nucleophilic attack models (pathway 2).

The reaction was studied using the stable, crystallographically characterised^[^ ^45^ ^]^ cation [(P^N)AuPh(OTf)]^+^ (**E**) as zero a point (Scheme 3). Geometries for the expected intermediates and transition states were easily located. All Au(III) species feature square‐planar four‐coordinate structures. Displacement of the triflate ligand to yield the Au(III) alkene dication **F** is strongly endergonic (by 20–25 kcal/mol) and not very sensitive to ligand variation. Displacement of tosylate (OTs^‐^) by olefin is even more endergonic (by about 10 kcal/mol), reflecting the higher nucleophilicity of OTs^−^_._

The structures of the alkene species **F** and **Z** deserve some discussion. Whereas mono‐cationic alkene and diene complexes could be isolated,^[^ ^20^, ^22^ ^]^ dicationic gold(III) alkene complexes are unknown. Nevertheless, their involvement in these reactions is a necessary postulate. **F** / **Z** is formed by triflate displacement from the OTf complex **E** (equ. 1) The precise mechanism for this step is unknown. Our search for a three‐coordinate “truly naked” intermediate has so far been unsuccessful, and an associative interchange mechanism^[^ ^61^ ^]^ is most likely.


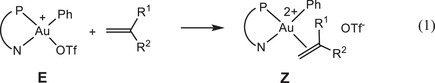


Structures of alkene complexes have been calculated without (**F**) and with (**Z**) inclusion of a triflate anion. The relative positions of the cation and triflate anion are subject to uncertainty, and the anion is unlikely to remain fixed in further reactions, so the energies of **Z** should be regarded as upper limits.

**Figure 1:**
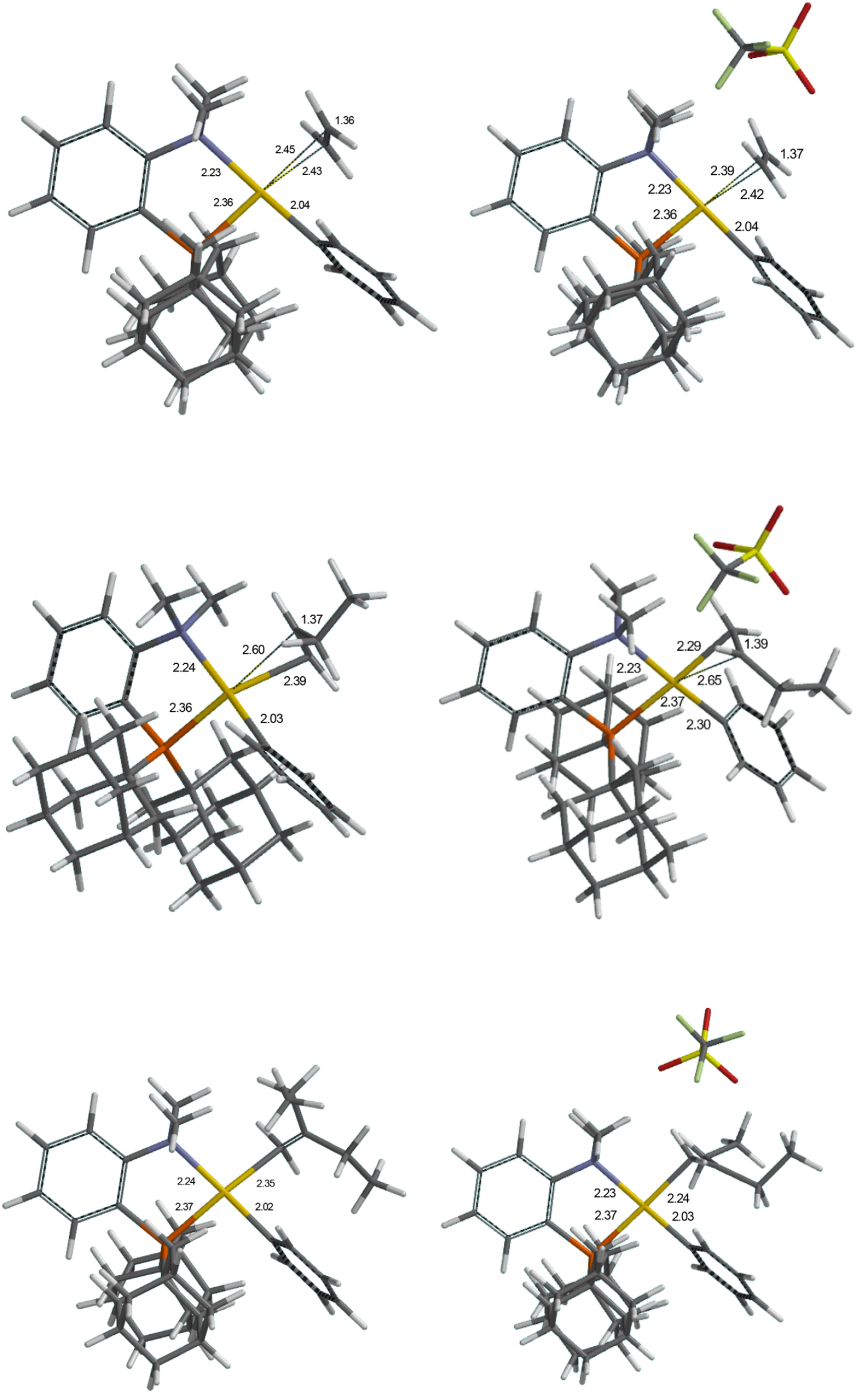
Optimized structure of alkene adducts **F** in the absence of a counteranion, and structures **Z** including one OTf^−^ anion, for ethene (top), 1‐butene (middle row), and 2‐ethyl‐1‐butene (bottom). Atom labels: P (orange), N (blue), Au (yellow), O (red), F (light green).

The calculated structures are shown in Figure 1. Not unexpectedly, the coordination mode of the alkene changes with the degree of substitution. For ethylene, the Au─C(alkene) bonds are nearly identical, and both C atoms carry a partial negative charge. In the 1‐butene adduct, coordination induces a strong difference between the negatively charged C1 and the more positively charged C2. In the 2‐ethyl‐1‐butene adduct finally, the Au‐C2 distance becomes so long (≥2.75 Å) as to be nonbonding, giving an η^1^‐alkene adduct where the C2 atom carries a substantial positive charge. Inclusion of OTf^−^ in structures **Z** reinforces the polarity between C1 and C2 and shortens the Au‐C1 bond lengths. This polarization facilitates nucleophilic attack. Pertinent calculated bond lengths for **F** and **Z** are collected in Table 1.

**Table 1:** Comparison of pertinent bond lengths (Å) for ethene, 1‐butene and 2‐ethyl‐1‐butene adducts **F**, for the smaller ligand L^Me^ = 1,2‐C_6_H_4_NMe_2_(PMe_2_) and full model L^Ad^ = 1,2‐C_6_H_4_NMe_2_(PAd_2_).

	alkene	M‐P	M‐N	M‐Ph	M‐C1	M‐C2
L^Me^	ethene	2.29	2.27	2.04	2.45	2.42
L^Me^	1‐butene	2.30	2.25	2.03	2.36	2.50
L^Me^	2‐ethylbutene	2.31	2.27	2.02	2.29	2.75
L^Ad^	ethene	2.36	2.23	2.04	2.43	2.45
L^Ad^	1‐butene	2.36	2.24	2.03	2.39	2.60
L^Ad^	2‐ethylbutene	2.36	2.23	2.04	2.35	>2.75

**Figure 2:**
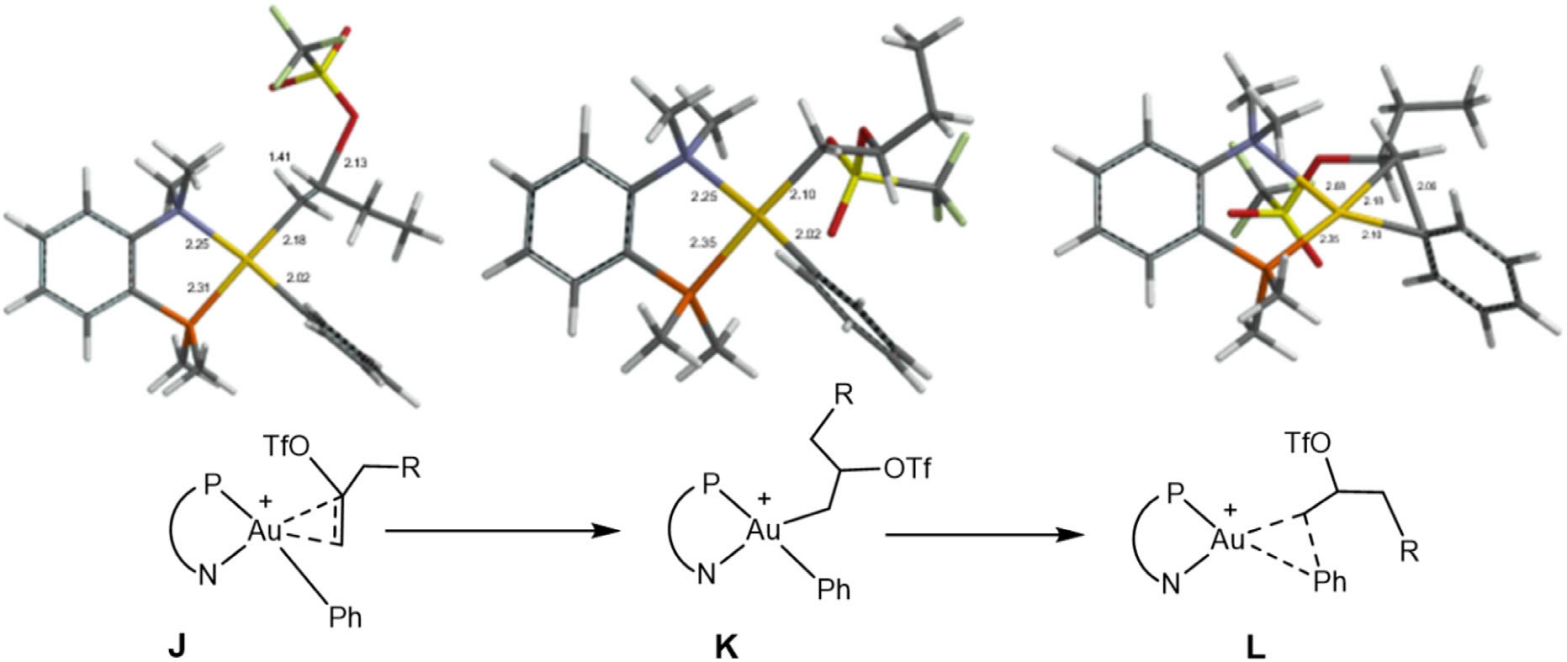
Structures of **J**, **K** and the reductive elimination transition states **L**, shown here for the L^Me^ ligand for clarity (atom labels: P orange, N blue).

The reaction scheme studied (Scheme 3) is only part of the complete reaction path, but it covers all steps required to determine the (regio‐)selectivity. The three key steps are summarized, for all variations, in Table 2. Geometries of the important transition states are shown in Figure 2 for the smaller L^Me^ rather than the L^Ad^ model for greater clarity.

**Table 2:** Gibbs free energies (kcal/mol) of key transition states for the insertion and the nucleophilic addition pathways.

Entry	F	G	J	L
1	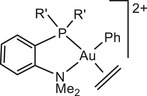 R' = H	36.3	16.9	28.2
2	R' = Me	39.6	19.2	35.5
3	R' = 1‐adamantyl	38.0	18.3	28.2
4	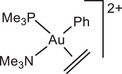	34.4	17.9	32.2
5	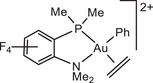	38.7	18.3	30.3
6	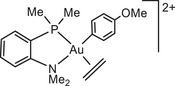	36.2	18.7	35.8
7	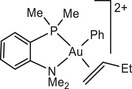	38.5	17.2	36.3
8	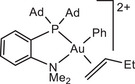	35.1	15.8	29.1
9	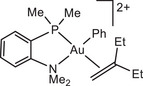	37.9	16.2	45.0
10	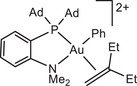	32.7	16.9	34.4
11	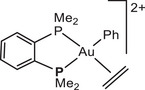	28.1	13.5	42.2

As outlined in Scheme 3, starting from the alkene complex **F** two pathways can be envisaged. In **Pathway 1,** the first reaction step is the migratory insertion of an alkene ligand into the Au‐aryl bond of a hypothetical dication [LAu(aryl)(alkene)]^2+^ (**F**), via transition state **G**, followed by Au‐aralkyl formation in **H** → **I**. These calculations replicate the model by Gandon and Patil^[^ ^41^ ^]^ and at this stage do not include the OTf^−^ anion. The optimized geometries relevant to the migratory olefin insertion step are very similar to the ones reported by Patil for the same system.

The preferred reaction path has the olefin coming in *cis* to the phosphine part of the P^N ligand. This results in a sizeable barrier at **G**, of the order of 10–15 kcal/mol above **F**. Even for the preferred path, the insertion barrier is larger than one might expect for this standard elementary step, and this can be attributed to the large *trans*‐influence that opposes insertion from the *cis* isomer. The alternative orientation has an even higher barrier. For the basic system, both orientations were checked. For the variations studied here, a few representative examples were explored. For this geometry, the effective barrier is even higher because the reactant structure already suffers from the full *trans*‐influence. For 1‐butene, the DFT results predict a clear preference for 1,2‐insertion.

**Pathway 2** assumes the same dication [LAu(aryl)(alkene)]^2+^ **F** as a starting point, but the first step in this case is a nucleophilic attack by the triflate anion on the coordinated olefin to give **J**. This is then followed by reductive C(aryl)‐C(alkyl) coupling, illustrated by structures **F** → **Z** → **J** → **K** → **L**. In this sequence, one OTf^‐^ anion is included as a co‐reagent. DFT produces very low barriers for the nucleophilic attack of the triflate anion on the coordinated olefin, between 0 and ∼5 kcal/mol.

The relevant transition states that determine the course of the whole reaction are **G** for insertion, **J** for external nucleophilic attack on the coordinated olefin, and **L** for the C(aryl)‐C(alkyl) reductive elimination step.

**Figure 3:**
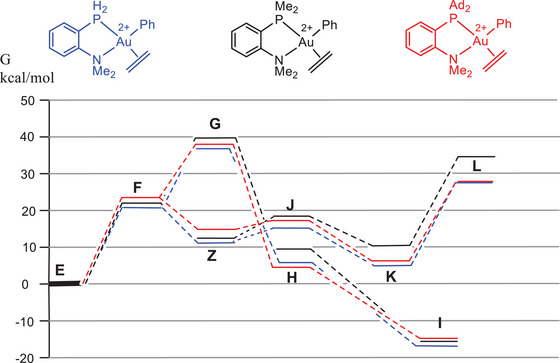
Energy trends for pathways 1 (alkene insertion, **F** → **G** → **H** → **I**) and 2 (nucleophilic attack, **F** → **Z** → **J** → **K** → **L**) as a function of steric hindrance in the P^N ligand.

Increasing steric hindrance in the sequence L^H^ < L^Me^ < L^Ad^ has little influence on the overall trends in all relevant transition states, as exemplified in Figure 3 for chelating P^N ligands (Table 2, entries 1–3). The same is true going from chelating P^N to nonchelated P, N structures. Compared to a complex with monodentate PMe_3_ and NMe_3_ ligands and using ethylene as a substrate, chelate formation increases the migratory insertion barrier. It also increases the barrier of reductive C─C coupling but by a smaller amount (Table 2, entry 4). Replacing the four aromatic hydrogens of L^Me^ by fluorines destabilizes the C─C coupling path (TS **L**) (Table 2, entry 5), leading to a lowering of the reductive elimination barrier by 5 kcal/mol. This is one of the larger ligand effects in the series studied here, and suggests that somewhat more weakly binding ligands could be an interesting target.

The importance of the counterion in determining the reaction path was checked by comparison with results for the tosylate (*p*‐MeC_6_H_4_SO_3_^−^) anion. This makes nucleophilic attack more difficult by about 5 kcal/mol, but insertion suffers even more (by 10 kcal/mol).

**Figure 4:**
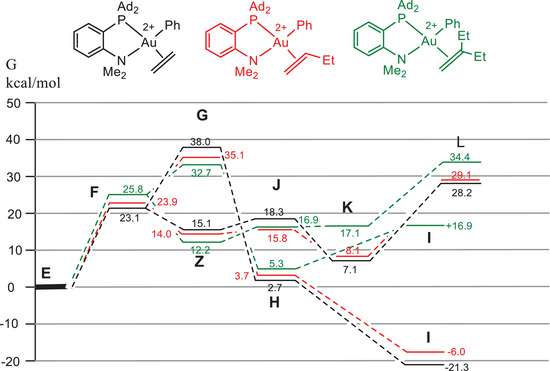
Energy trends in the L^Ad^ system as a function of 1‐alkene structure.

The effect of olefin variation (butene/hexene instead of ethene) is similarly small, and changing the alkene from ethylene to 1‐butene has little effect on the overall trend. Only for 2‐ethyl‐1‐butene, which was not experimentally investigated, is there a suggestion that the final aryl‐alkyl elimination step **L** may become higher than the 1,2‐insertion barrier **G** (Figure 4), although here the difference is slight and of the order of computational uncertainty. For the L^Me^ complex of 2‐ethyl‐1‐butene we calculate a much more difficult reductive elimination step, by nearly 10 kcal/mol, while the olefin insertion barrier remains at its L^Ad^ value. The result could be indicative of a switch in mechanism; however, we have to keep in mind that the step between L^Me^ and the full L^Ad^ model would have to be substantial, about 7 kcal/mol in favor of the insertion path, which leads to nearly perfectly matched barriers for insertion and nucleophilic attack.

**Figure 5:**
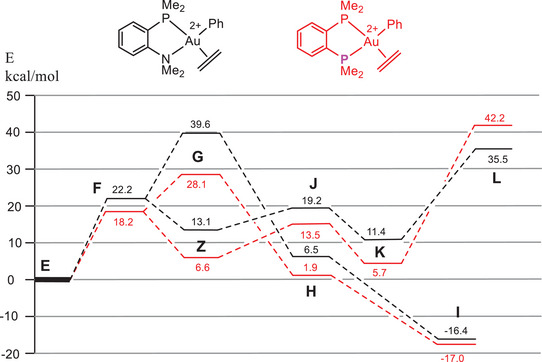
Comparison of P^N and P^P energy profiles, with ethylene as substrate.

By contrast, going from P^N to a P^P donor, which places a strong *trans*‐effect phosphine donor *trans* to the phenyl ligand, results in a major reduction in the migratory insertion barrier **G**. There is no longer such a strong *trans*‐influence blocking insertion, while at the same time the reductive elimination barrier **L** increases by 7 kcal/mol (Figure 5). This N → P variation, that is, where both gold coordination sites are occupied by electron‐rich P donors, would completely change the chemistry of the complex, and it seems likely that migratory insertion may be preferred. The effect of the P^P ligand therefore mirrors the reactivity of Bourissou's even more strongly electron‐donating P^C^(‐)^ chelate ligand referred to above.^[^ ^35^, ^36^, ^37^, ^38^, ^39^ ^]^

For the migratory pathway, consideration of the regiochemistry is important. 1‐Alkenes may undergo 1,2‐insertion, leading to linear gold alkyls of type **I**, or insert in 2,1‐fashion, giving rise to branched gold alkyls. As pointed out above, the Pd‐catalyzed production of Heck‐type arylated olefins requires 2,1‐insertion to explain the formation of 1‐aryl‐1‐alkenes (Scheme 1b). However, calculations show that for the gold complexes under investigation, in all cases 2,1‐insertion encounters a higher barrier, which increases with increasing steric hindrance, from 4.7 kcal/mol for L^Me^ and 1‐butene to 20.0 kcal/mol for L^Ad^ and 2‐ethyl‐1‐butene. Product release from linear gold alkyls **I** by β‐H elimination would generate terminal 1‐alkenes, which is not observed experimentally. The calculated alkene 1,2‐insertion preference therefore cannot explain the formation of Heck‐type alkenes of type **A** by gold catalysts; of course, this regioselectivity rules out a migratory insertion pathway.

## 3 Conclusions

Ligands are the toolbox of homogeneous catalysis. While elementary steps may be understood in principle, there often remains uncertainty about the different roles of various aspects of ligand structures. The present case, centred on P^N ligands, illustrates in a more quantitative fashion the contribution of important ligand features. Energetically competitive species with a dangling rather than coordinated ‐NMe_2_ donor could not be identified, and the chelate structure prevails in all cases. The postulated dicationic alkene adducts [(P^N)AuPh(CH_2 _═CR^1^R^2^)]^2+^ show unequal bonding to the alkene C‐atoms which increases with increased steric hindrance and with inclusion of the OTf counteranion in the model, commensurate with increasing carbocationic character of C2. While sterics (1‐adamantyl vs. methyl), electronics (P^P vs. P^N), rigidity (nonchelating P|N vs. P^N chelate) and anion nucleophilicity (OTf^‐^ vs. OTs^‐^) all have an effect, the preferred reaction pathway is largely directed by the relative *trans*‐influence of the N and P donors of the chelate ligand, which favors an external nucleophilic attack on the coordinated olefin over migratory insertion of the alkene into the Au‐aryl bond. The barriers of this nucleophilic attack are very low. The calculations are in agreement with the experimental observation of an alkyl triflate ester as the primary organic product.^[^ ^45^ ^]^ Although the triflate anion is often regarded as nonnucleophilic, its nucleophilicity is in fact rather variable, depending on the solvent, but certainly nonnegligeable, and numerous examples of triflate as potent nucleophile have been reported.^[^ ^62^ ^]^ The triflate ester formation step en‐route to the generation of Heck‐type benzyl alkenes of type **A** therefore parallels the well‐known gold‐catalyzed alkene heteroarylation,^[^ ^63^ ^]^ a process that typically involves stronger nucleophiles such as alcohols or amines. Intra‐ and intermolecular alkene heteroarylations have been the subject of a number of theoretical studies^[^ ^51^, ^64^ ^]^ which have resulted in rather similar reaction profiles. The present work extends these reaction principles to very weak nucleophiles such as the triflate anion, a testimony of the extreme electrophilicity of dicationic gold(III) alkene adducts.

## Conflict of Interest

The authors declare no conflict of interest.

## Supporting information



Supporting Information

## Data Availability

The data that support the findings of this study are available in the supplementary material of this article.

## References

[1] A. S. K. Hashmi , Gold Bull 2004, 37, 51.

[2] A. S. K. Hashmi , G. J. Hutchings , Angew. Chem. Int. Ed. 2006, 45, 7896. 10.1002/anie.200602454 17131371

[3] A. S. K. Hashmi , F. D. Toste , Eds.: Modern Gold‐Catalyzed Synthesis, Wiley‐VCH, Weinheim, 2012.

[4] F. D. Toste , V. Michelet , (eds): Gold Catalysis – a Homogeneous Approach, Imperial College Press, London, 2014.

[5] L. M. Slaughter , (ed.): Homogeneous Gold Catalysis, Springer, Heidelberg, 2015.

[6] Chem. Rev. 2008, 108 Themed issue: Coinage Metals in Organic Synthesis, pp. 2793–3442. 18698732 10.1021/cr800415x

[7] Acc. Chem. Res. 2014, 47 Themed issue: Gold Catalysis. 10.1021/ar5000506 24635456

[8] Chem. Soc. Rev. 2016, 45 Themed issue: Coinage Metals in Organic Synthesis, pp. 4445–4627. 27430042 10.1039/c6cs90072k

[9] Chem. Rev. 2021, 121 (14), Themed issue: Gold chemistry, pp. 8309–9164. 34315211 10.1021/acs.chemrev.1c00393

[10] B. Huang , M. Hu , F. D. Toste , Trends Chem. 2020, 2, 707. 34341775 10.1016/j.trechm.2020.04.012 PMC8321390

[11] J. F. Hartwig , Organotransition Metal Chemistry From Bonding to Catalysis, University Science Books, Sausalito, 2010.

[12] M. Bochmann , Organometallics and Catalysis: An Introduction, Oxford University Press, Oxford, 2014.

[13] R. H. Crabtree , The Organometallic Chemistry of the Transition Metals, 7th ed., Wiley 2019.

[14] M. Joost , A. Amgoune , D. Bourissou , Angew. Chem. Int. Ed. 2015, 54, 15022. 10.1002/anie.201506271 26768342

[15] L. Rocchigiani , M. Bochmann , Chem. Rev. 2021, 121, 8364. 32966741 10.1021/acs.chemrev.0c00552

[16] J. Monot , E. Marelli , B. Martin‐Vaca , D. Bourissou , Chem. Soc. Rev. 2023, 3543. 37129171 10.1039/d2cs00564f

[17] P. Font , H. Valdés , X. Ribas , Angew. Chem. Int. Ed. 2024, 63, e202405824. 10.1002/anie.202405824 38687322

[18] M. S. Martinsen Holmsen , A. Nova , M. Tilset , Acc. Chem. Res. 2023, 56, 3654. 38051910 10.1021/acs.accounts.3c00595 PMC10734256

[19] D.‐A. Roşca , J. A. Wright , M. Bochmann , Dalton Trans. 2015, 44, 20785. 26584519 10.1039/c5dt03930d PMC4669034

[20] (a) N. Savjani , D.‐A. Roşca , M. Schormann , M. Bochmann , Angew. Chem. Int. Ed. 2013, 52, 874. 10.1002/anie.201208356 23180688

[21] L. Rocchigiani , J. Fernandez‐Cestau , G. Agonigi , I. Chambrier , P. H. M. Budzelaar , M. Bochmann , Angew. Chem. Int. Ed. 2017, 56, 13861. 10.1002/anie.201708640 PMC5698712 28892244

[22] (a) E. Langseth , M. L. Scheuermann , D. Balcells , W. Kaminsky , K. I. Goldberg , O. Eisenstein , R. H. Heyn , M. Tilset , Angew. Chem. Int. Ed. 2013, 52, 1660. 10.1002/anie.201209140 23283802

[23] D.‐A. Roşca , D. A. Smith , D. L. Hughes , M. Bochmann , Angew. Chem. Int. Ed. 2012, 51, 10643. 10.1002/anie.201206468 22997099

[24] D.‐A. Roşca , J. A. Wright , D. L. Hughes , M. Bochmann , Nat. Commun. 2013, 4, 2167. 10.1038/ncomms3167 23852042

[25] D.‐A. Roşca , J. Fernandez‐Cestau , J. Morris , J. A. Wright , M. Bochmann , Science Adv. 2015, 1, e1500761. 10.1126/sciadv.1500761 PMC4646827 26601313

[26] L. Rocchigiani , J. Fernandez‐Cestau , I. Chambrier , P. Hrobarik , M. Bochmann , J. Am. Chem. Soc. 2018, 140, 8287. 29860842 10.1021/jacs.8b04478 PMC6047844

[27] L. Rocchigiani , W. T. Klooster , S. J. Coles , D. L. Hughes , P. Hrobarik , M. Bochmann , Chem. Eur. J. 2020, 26, 8267. 32101346 10.1002/chem.202000016

[28] E. Y. Tsui , P. Müller , J. P. Sadighi , Angew. Chem. Int. Ed. 2008, 47, 8937. 10.1002/anie.200803842 18855961

[29] D.‐A. Roşca , J. Fernandez‐Cestau , D. L. Hughes , M. Bochmann , Organometallics 2015, 34, 2098. 26146435 10.1021/om501165z PMC4482408

[30] R. Kumar , J.‐P. Krieger , E. Gómez‐Bengoa , T. Fox , A. Linden , C. Nevado , Angew. Chem. Int. Ed. 2017, 56, 12862. 10.1002/anie.201705557 28902956

[31] A. Pintus , L. Rocchigiani , J. Fernandez‐Cestau , P. H. M. Budzelaar , M. Bochmann , Angew. Chem. Int. Ed. 2016, 55, 12321. 10.1002/anie.201607522 PMC5113781 27592697

[32] A. Pintus , M. Bochmann , RSC Adv. 2018, 8, 2795. 35541449 10.1039/c7ra13481a PMC9077454

[33] J. Fernandez‐Cestau , L. Rocchigiani , A. Pintus , R. J. Rama , P. H. M. Budzelaar , M. Bochmann , Chem. Commun 2018, 54, 11447. 10.1039/c8cc06409a 30251719

[34] Another bimolecular alkyne hydroauration mechanism involving Au(I)-Au(III) binuclear species has recently been proposed: J. Martin , J. Schorgenhumer , C. Nevado , JACS Au 2025, 5, 1439. 40151240 10.1021/jacsau.5c00056 PMC11938007

[35] F. Rekhroukh , R. Brousses , A. Amgoune , D. Bourissou , Angew. Chem. Int. Ed. 2015, 54, 1266. 10.1002/anie.201409604 25353964

[36] F. Rekhroukh , L. Estevez , C. Bijani , K. Miqueu , A. Amgoune , D. Bourissou , Organometallics 2016, 35, 995. 10.1002/anie.201511111 26833571

[37] J. Monot , E. Marelli , B. Martin‐Vaca , D. Bourissou , Chem. Soc. Rev. 2023, 52, 3543. 37129171 10.1039/d2cs00564f

[38] F. Rekhroukh , C. Blons , L. Estévez , S. Mallet‐Ladeira , K. Miqueu , A. Amgoune , D. Bourissou , Chem. Sci. 2017, 8, 4539. 28660067 10.1039/c7sc00145b PMC5472032

[39] F. Rekhroukh , L. Estevez , S. Mallet‐Ladeira , K. Miqueu , A. Amgoune , D. Bourissou , J. Am. Chem. Soc. 2016, 138, 11920. 27533923 10.1021/jacs.6b07035

[40] J. Serra , P. Font , P.; E. D. Sosa Carrizo , S. Mallet‐Ladeira , S. Massou , T. Parella , K. Miqueu , A. Amgoune , X. Ribas , D. Bourissou , Chem. Sci. 2018, 9, 3932. 29780525 10.1039/c7sc04899h PMC5941201

[41] V. W. Bhoyare , E. D. Sosa Carrizo , C. C. Chintawar , V. Gandon , N. T. Patil , J. Am. Chem. Soc. 2023, 145, 8810. 37061943 10.1021/jacs.3c02544

[42] V. W. Bhoyare , A. G. Tathe , V. Gandon , N. T. Patil , Angew. Chem. 2023, e202312786. 37779346 10.1002/anie.202312786

[43] R. F. Heck , Acc. Chem. Res. 1979, 12, 146.

[44] (a) For computational modelling of palladium‐catalyzed Heck reactions see for example: H. von Schenck , B. Åkermark , M. Svensson , Organometallics 2002, 21, 2248.

[45] P. H. M. Budzelaar , M. Bochmann , M. Landrini , L. Rocchigiani , Angew. Chem. Int. Ed. 2024, e202317774. 10.1002/anie.202317774 38695675

[46] J. Rodriguez , K. Miqueu , A. Zeineddine , E. D. Sosa Carrizo , N. Saffon‐Merceron , A. Amgoune , D. Bourissou , Chem. Sci. 2019, 10, 7183. 31588286 10.1039/c9sc01954e PMC6685352

[47] M. Navarro , A. Toledo , S. Mallet‐Ladeira , E. D. Sosa , K. Miqueu , D. Bourissou , Chem. Sci. 2020, 11, 2750. 34084334 10.1039/c9sc06398f PMC8157524

[48] J. Rodriguez , A. Tabey , S. Mallet‐Ladeira , D. Bourissou , Chem. Sci. 2021, 12, 7706. 34168822 10.1039/d1sc01483h PMC8188461

[49] M. Navarro , A. Tabey , G. Szalóki , S. Mallet‐Ladeira , D. Bourissou , Organometallics 2021, 40, 1571.

[50] J. A. Cadge , P. J. Gates , J. F. Bower , C. A. Russell , J. Am. Chem. Soc. 2022, 144, 19719. 36282061 10.1021/jacs.2c10432 PMC9634805

[51] K. Muratov , E. Zaripov , M. V. Berezovski , F. Gagosz , J. Am. Chem. Soc. 2024, 146, 3660. 38315643 10.1021/jacs.3c08943

[52] A. Zeineddine , L. Estevez , S. Mallet‐Ladeira , K. Miqueu , A. Amgoune , D. Bourissou , Nat. Commun. 2017, 8, 565. 28924193 10.1038/s41467-017-00672-8 PMC5603523

[53] M. Joost , A. Zeineddine , L. Estévez , S. Mallet‐Ladeira , K. Miqueu , A. Amgoune , D. Bourissou , J. Am. Chem. Soc. 2014, 136, 14654. 25268830 10.1021/ja506978c

[54] A reviewer requested the inclusion of a more specific comment on the Reply by Patil et al. to our mechanistic investigation^[45]^: V. W. Bhoyare , E. D. Sosa Carrizo , V. Gandon , N. T. Patil , Angew. Chem. Int. Ed. 2024, e202411948. These authors did not provide substantial arguments against our proposal, yet concluded “Our results suggest that the product formation solely cannot be rationalized based on hetero‐arylation of alkenes under ligand‐enabled Au(I)/Au(III) catalysis” (where “solely” appears intended to hint at unspecified alternatives). While such statement seems designed to distract from the acceptation in principle of the heteroarylation mechanism, the overall conclusion is positive: “…we can only commend Budzelaar et al. for their foresight in developing this interesting scenario, which we believe will be very fruitful in further advancing gold catalysis”.

[55] M. J. Frisch , G. W. Trucks , H. B. Schlegel , G. E. Scuseria , M. A. Robb , J. R. Cheeseman , G. Scalmani , V. Barone , G. A. Petersson , H. Nakatsuji , X. Li , M. Caricato , A. V. Marenich , J. Bloino , B. G. Janesko , R. Gomperts , B. Mennucci , H. P. Hratchian , J. V. Ortiz , A. F. Izmaylov , J. L. Sonnenberg , Williams‐Young , F. D. Ding , F. Lipparini , F. Egidi , J. Goings , B. Peng , A. Petrone , T. Henderson , D. Ranasinghe , V. G. Zakrzewski , J. Gao , N. Rega , G. Zheng , W. Liang , M. Hada , M. Ehara , K. Toyota , R. Fukuda , J. Hasegawa , M. Ishida , T. Nakajima , Y. Honda , O. Kitao , H. Nakai , T. Vreven , K. Throssell , J. A. Montgomery Jr. , J. E. Peralta , F. Ogliaro , M. J. Bearpark , J. J. Heyd , E. N. Brothers , K. N. Kudin , V. N. Staroverov , T. A. Keith , R. Kobayashi , J. Normand , K. Raghavachari , A. P. Rendell , J. C. Burant , S. S. Iyengar , J. Tomasi , M. Cossi , J. M. Millam , M. Klene , C. Adamo , R. Cammi , J. W. Ochterski , R. L. Martin , K. Morokuma , O. Farkas , J. B. Foresman , D. J. Fox, C.01 ed., Gaussian, Inc., Wallingford CT, 2016.

[56] (a) J. Baker , 2.4 ed., Parallel Quantum Solutions, Fayetteville, AR, 2001;

[57] H. Y. S. Yu , X. He , S. H. L. Li , D. G. Truhlar , Chem. Sci. 2016, 7, 5032. 30155154 10.1039/c6sc00705h PMC6018516

[58] D. Figgen , K. A. Peterson , M. Dolg , H. Stoll , J. Chem. Phys. 2009, 130, 164108. 19405562 10.1063/1.3119665

[59] B. P. Pritchard , D. Altarawy , B. Didier , T. D. Gibson , T. L. Windus , J. Chem. Inf. Model. 2019, 59, 4814. 31600445 10.1021/acs.jcim.9b00725

[60] (a) J. B. Foresman , T. A. Keith , K. B. Wiberg , J. Snoonian , M. J. Frisch , J. Phys. Chem. 1996, 100, 16098;

[61] (a) C. H. Langford , H. B. Gray , Ligand Substitution Processes, W. A. Benjamin, New York, 1965.

[62] The nucleophilicity of the triflate anion is variable depending on the solvent but non‐negligeable: B. Dhakal , L. Bohé , D. Crich , J. Org. Chem. 2017, 82, 9263. 28858509 10.1021/acs.joc.7b01850 PMC5600715

[63] (a) G. Zhang , L. Cui , Y. Wang , L. Zhang , J. Am. Chem. Soc. 2010, 132, 1474. 20050647 10.1021/ja909555d

[64] (a) M. Rigoulet , K. Miqueu , D. Bourissou , Chem. Eur. J. 2022, e202202110. 35876716 10.1002/chem.202202110 PMC9805180

