# Supplementary material for: Nucleophilic Addition versus Migratory Insertion Pathways in the Gold‐Catalyzed Heck Reaction: A Computational Study
Source: Chemistry. 2025 Jul 7;31(41):e202501645. doi: 10.1002/chem.202501645 (PMC12284628; doi:10.1002/chem.202501645)
Supplement: Supplementary file 1 — Supporting Information [file CHEM-31-e202501645-s001.pdf]

**Nucleophilic Addition *versus* Migratory Insertion Pathways in the Gold-Catalysed Heck Reaction: A Computational Study**

**Supporting Information**

***Methods***

All calculations were done using Gaussian-16 (rev C01)[1] in combination with an external optimizer.[2] Structures were fully optimized, without symmetry restrictions, using the dispersion-aware MN15 functional,[3] the cc-pVDZ(pp)[4] basis set[5] on all atoms, and the SMD continuum solvation model for dichloromethane[6] using the explicitly selected "non-symmetric" algorithm (keyword NonSymmetricPCM).[7] All stationary points were verified (through vibrational analysis) to be minima (no negative Hessian eigenvalues) or transition states (exactly one negative eigenvalue corresponding to the correct reaction).

IRC calculations were mostly unsuccessful. When run with default settings, many indicated failure. It is possible that the combination with solvation corrections plays a role here. In any case, the only way to avoid failure messages was to turn off all checks, which makes IRC basically meaningless. In cases where there was doubt about the nature of transition states, they were checked by moving each TS along the reaction coordinate (in both directions, 0.03 to 0.20 Bohr) and re-optimizing. Improved total energies were obtained from single-point calculations at the optimized geometries, using the cc-pVTZ(pp) basis set,[8] and these energies were combined with thermal corrections (enthalpy and entropy, 298 K) to obtain the final Gibbs free energies mentioned in the text. Entropy corrections were scaled by 0.67 to account for reduced freedom in solution.[9] Relevant total and relative energies are included in Table S1, and a more compact summary showing only relevant relative free energies (corrrespondng to the free energy profiles) can be found in Table S2. There are a few features that we believe result in larger error margins than usual for this kind of study, as already noted in our letter.

[1] M. J. Frisch, G. W. Trucks, H. B. Schlegel, G. E. Scuseria, M. A. Robb, J. R. Cheeseman, G. Scalmani, V. Barone, G. A. Petersson, H. Nakatsuji, X. Li, M. Caricato, A. V. Marenich, J. Bloino, B. G. Janesko, R. Gomperts, B. Mennucci, H. P. Hratchian, J. V. Ortiz, A. F. Izmaylov, J. L. Sonnenberg, Williams-Young, F. D.; Ding, F. Lipparini, F. Egidi, J. Goings, B. Peng, A. Petrone, T. Henderson, D. Ranasinghe, V. G. Zakrzewski, J. Gao, N. Rega, G. Zheng, W. Liang, M. Hada, M. Ehara, K. Toyota, R. Fukuda, J. Hasegawa, M. Ishida, T. Nakajima, Y. Honda, O. Kitao, H. Nakai, T. Vreven, K. Throssell, J. A. Montgomery, Jr., J. E. Peralta, F. Ogliaro, M. J. Bearpark, J. J. Heyd, E. N. Brothers, K. N. Kudin, V. N. Staroverov, T. A. Keith, R. Kobayashi, J. Normand, K. Raghavachari, A. P. Rendell, J. C. Burant, S. S. Iyengar, J. Tomasi, M. Cossi, J. M. Millam, M. Klene, C.

Adamo, R. Cammi, J. W. Ochterski, R. L. Martin, K. Morokuma, O. Farkas, J. B. Foresman, D. J. Fox, C.01 ed., Gaussian, Inc., , Wallingford CT, **2016**.

[2] (a) J. Baker, 2.4 ed., Parallel Quantum Solutions, Fayetteville, AR, **2001**; (b) J. Baker, *J. Comput. Chem.* **1986**, *7*, 385-395; (c) P. H. M. Budzelaar, *J. Comput. Chem.* **2007**, *28*, 2226-2236.

[3] H. Y. S. Yu, X. He, S. H. L. Li, D. G. Truhlar, *Chem. Sci.* **2016**, *7*, 5032-5051.

[4] D. Figgen, K. A. Peterson, M. Dolg, H. Stoll, *J. Chem. Phys.* **2009**, *130*, 164108.

[5] B. P. Pritchard, D. Altarawy, B. Didier, T. D. Gibson, T. L. Windus, *J. Chem. Inf. Model.* **2019**, *59*, 4814-4820.

[6] (a) J. B. Foresman, T. A. Keith, K. B. Wiberg, J. Snoonian, M. J. Frisch, *J. Phys. Chem.* **1996**, *100*, 16098-16104; (b) A. V. Marenich, C. J. Cramer, D. G. Truhlar, *J. Phys. Chem. B* **2009**, *113*, 6378-6396.

[7] P. H. M. Budzelaar, M. Bochmann, M. Landrini, L. Rocchigiani, *Angew. Chem. Int. Ed.* **2024**, e202317774.

[8] F. Weigend, R. Ahlrichs, *Phys. Chem. Chem. Phys.* **2005**, *7*, 3297-3305.

[9] (a) R. Raucoules, T. de Bruin, P. Raybaud, C. Adamo, *Organometallics* **2009**, *28*, 5358-5367; (b) S. Tobisch, T. Ziegler, *J. Am. Chem. Soc.* **2004**, *126*, 9059-9071.

*Table S1. Total (h) and relative (kcal/mol) energies*

| ***Name*** | ***Formula*** | ***E SV(P)*** | ***Hcorr*** | ***TScorr*** | ***E TZVP*** | ***G*** | ***on scale*** | ***Grel*** |
|---|---|---|---|---|---|---|---|---|
| **refs** | | | | | | | | |
| Eth | C2H4 | -78.40686 | 0.05500 | 0.02487 | -78.50800 | -78.46966 | | |
| Btn | C4H8 | -156.86432 | 0.11439 | 0.03378 | -157.06182 | -156.97006 | | |
| Ebn | C6H12 | -235.32104 | 0.17358 | 0.03911 | -235.61509 | -235.46771 | | |
| OTf- | CF3O3S | -960.35980 | 0.03646 | 0.04057 | -961.26462 | -961.25534 | | |
| OTs- | C7H7O3S | -893.66811 | 0.14253 | 0.04811 | -894.48381 | -894.37352 | | |
| NMe3 | C3H9N | -174.08749 | 0.12637 | 0.03372 | -174.30812 | -174.20433 | | |
| **PMe2** | | | | | | | | |
| LAu_E_1+_Ph_OTf_rot4 | C17H21AuF3NO3PS | -2112.41787 | 0.39392 | 0.09057 | -2114.25917 | -2113.92594 | -1152.67059 | 0.00 |
| LAu_F_2+_Ph_Eth_rot1 | C18H25AuNP | -1230.41292 | 0.41427 | 0.07494 | -1231.46896 | -1231.10491 | -1152.63524 | 22.18 |
| LAu_G_2+_Ph_Eth_insTS | C18H25AuNP | -1230.38523 | 0.41214 | 0.07306 | -1231.44037 | -1231.07718 | -1152.60751 | 39.58 |
| LAu_H_2+_CH2CH2Ph_ortho | C18H25AuNP | -1230.44501 | 0.41554 | 0.07360 | -1231.49609 | -1231.12986 | -1152.66019 | 6.53 |
| LAu_I_1+_CH2CH2Ph_OTf | C19H25AuF3NO3PS | -2190.87423 | 0.45452 | 0.09621 | -2192.81179 | -2192.42173 | -1152.69672 | -16.40 |
| LAu_J_1+_Ph_EthOTf_extTS | C19H25AuF3NO3PS | -2190.80314 | 0.45152 | 0.09575 | -2192.75237 | -2192.36500 | -1152.63999 | 19.20 |
| LAu_K_1+_Ph_CH2CH2OTf | C19H25AuF3NO3PS | -2190.83471 | 0.45339 | 0.09609 | -2192.76872 | -2192.37971 | -1152.65470 | 9.97 |
| LAu_L_1+_Ph_CH2CH2OTf_rdelTS_rot4 | C19H25AuF3NO3PS | -2190.79952 | 0.45161 | 0.09561 | -2192.72662 | -2192.33907 | -1152.61406 | 35.48 |
| LAu_Z_1+_Ph_Eth__OTf | C19H25AuF3NO3PS | -2190.80820 | 0.45195 | 0.09926 | -2192.76014 | -2192.37470 | -1152.64969 | 13.12 |
| **PMe2/Btn** | | | | | | | | |
| Inherited | C17H21AuF3NO3PS | -2112.41787 | 0.39392 | 0.09057 | -2114.25917 | -2113.92594 | -1152.67059 | 0.00 |
| LAu_F_2+_Ph_Btna | C20H29AuNP | -1308.87397 | 0.47405 | 0.08153 | -1310.02621 | -1309.60679 | -1152.63672 | 21.25 |
| LAu_F_2+_Ph_Btnb | C20H29AuNP | -1308.87146 | 0.47380 | 0.08091 | -1310.02348 | -1309.60388 | -1152.63382 | 23.07 |
| LAu_G_2+_Ph_Btn_12insTS | C20H29AuNP | -1308.84642 | 0.47145 | 0.07919 | -1309.99765 | -1309.57926 | -1152.60920 | 38.52 |
| LAu_G_2+_Ph_Btn_21insTS | C20H29AuNP | -1308.84160 | 0.47216 | 0.07775 | -1309.99189 | -1309.57183 | -1152.60177 | 43.19 |
| LAu_H_2+_CH2CHEtPh_ortho_Y | C20H29AuNP | -1308.90056 | 0.47452 | 0.08053 | -1310.04827 | -1309.62771 | -1152.65765 | 8.12 |
| LAu_H_2+_CHEtCH2Ph_ortho_X | C20H29AuNP | -1308.89971 | 0.47503 | 0.08099 | -1310.04618 | -1309.62542 | -1152.65536 | 9.56 |
| LAu_I_1+_CH2CHEtPh_OTf_X | C21H29AuF3NO3PS | -2269.33062 | 0.51332 | 0.10318 | -2271.36294 | -2270.91875 | -1152.69334 | -14.28 |
| LAu_I_1+_CHEtCH2Ph_OTf_Y | C21H29AuF3NO3PS | -2269.33003 | 0.51305 | 0.10111 | -2271.36132 | -2270.91601 | -1152.69061 | -12.56 |

| ***Name*** | ***Formula*** | ***E SV(P)*** | ***Hcorr*** | ***TScorr*** | ***E TZVP*** | ***G*** | ***on scale*** | ***Grel*** |
|---|---|---|---|---|---|---|---|---|
| LAu_J_1+_Ph_BtnOTf_12extTS | C21H29AuF3NO3PS | -2269.26937 | 0.51034 | 0.10026 | -2271.31182 | -2270.86865 | -1152.64325 | 17.16 |
| LAu_J_1+_Ph_BtnOTf_21extTS | C21H29AuF3NO3PS | -2269.26087 | 0.51053 | 0.10118 | -2271.30256 | -2270.85982 | -1152.63441 | 22.70 |
| LAu_K_1+_Ph_CH2CHEtOTf_X | C21H29AuF3NO3PS | -2269.29421 | 0.51259 | 0.10133 | -2271.32459 | -2270.87989 | -1152.65449 | 10.11 |
| LAu_K_1+_Ph_CHEtCH2OTf_Y | C21H29AuF3NO3PS | -2269.28510 | 0.51263 | 0.10170 | -2271.31484 | -2270.87034 | -1152.64494 | 16.10 |
| LAu_L_1+_Ph_CH2CHEtOTf_rdelTS_Y | C21H29AuF3NO3PS | -2269.25794 | 0.51067 | 0.10084 | -2271.28121 | -2270.83810 | -1152.61270 | 36.33 |
| LAu_L_1+_Ph_CHEtCH2OTf_rdelTS_Y | C21H29AuF3NO3PS | -2269.24599 | 0.51119 | 0.10206 | -2271.26906 | -2270.82625 | -1152.60084 | 43.77 |
| LAu_Z_1+_Ph_Btn__OTf | C21H29AuF3NO3PS | -2269.27212 | 0.51108 | 0.10272 | -2271.31802 | -2270.87577 | -1152.65036 | 12.70 |
| **PMe2/Ebn** | | | | | | | | |
| ***Inherited*** | C17H21AuF3NO3PS | -2112.41787 | 0.39392 | 0.09057 | -2114.25917 | -2113.92594 | -1152.67059 | 0.00 |
| LAu_F_2+_Ph_Ebn | C22H33AuNP | -1387.32934 | 0.53356 | 0.08557 | -1388.57720 | -1388.10097 | -1152.63326 | 23.43 |
| LAu_G_2+_Ph_Ebn_12insTS | C22H33AuNP | -1387.30681 | 0.53071 | 0.08277 | -1388.55323 | -1388.07797 | -1152.61026 | 37.86 |
| LAu_G_2+_Ph_Ebn_21insTS | C22H33AuNP | -1387.29077 | 0.53175 | 0.08342 | -1388.53629 | -1388.06043 | -1152.59271 | 48.87 |
| LAu_H_2+_CEt2CH2Ph_ortho_X | C22H33AuNP | -1387.34987 | 0.53465 | 0.08412 | -1388.59096 | -1388.11267 | -1152.64496 | 16.09 |
| LAu_H_2+_CH2CEt2Ph_ortho_X | C22H33AuNP | -1387.35441 | 0.53329 | 0.08362 | -1388.59883 | -1388.12156 | -1152.65385 | 10.51 |
| LAu_I_1+_CEt2CH2Ph_OTf_X | C23H33AuF3NO3PS | -2347.76241 | 0.57332 | 0.10503 | -2349.88815 | -2349.38520 | -1152.66214 | 5.30 |
| LAu_I_1+_CH2CEt2Ph_OTf_X | C23H33AuF3NO3PS | -2347.77940 | 0.57217 | 0.10451 | -2349.90723 | -2349.40509 | -1152.68203 | -7.18 |
| LAu_J_1+_Ph_EbnOTf_12extTS | C23H33AuF3NO3PS | -2347.73007 | 0.56920 | 0.10563 | -2349.86594 | -2349.36751 | -1152.64446 | 16.40 |
| LAu_J_1+_Ph_EbnOTf_21extTS | C23H33AuF3NO3PS | -2347.72265 | 0.56975 | 0.10670 | -2349.85824 | -2349.35998 | -1152.63693 | 21.13 |
| LAu_K_1+_Ph_CEt2CH2OTf_X | C23H33AuF3NO3PS | -2347.71848 | 0.57224 | 0.10500 | -2349.84292 | -2349.34103 | -1152.61797 | 33.02 |
| LAu_K_1+_Ph_CH2CEt2OTf_X | C23H33AuF3NO3PS | -2347.74537 | 0.57145 | 0.10380 | -2349.86729 | -2349.36538 | -1152.64232 | 17.74 |
| LAu_L_1+_Ph_CEt2CH2OTf_rdelTS_X | C23H33AuF3NO3PS | -2347.68995 | 0.57038 | 0.10514 | -2349.80993 | -2349.30999 | -1152.58693 | 52.50 |
| LAu_L_1+_Ph_CH2CEt2OTf_rdelTS_X | C23H33AuF3NO3PS | -2347.70218 | 0.56944 | 0.10535 | -2349.82083 | -2349.32198 | -1152.59892 | 44.98 |
| LAu_Z_1+_Ph_Ebn__OTf | C23H33AuF3NO3PS | -2347.73273 | 0.56998 | 0.10812 | -2349.87345 | -2349.37591 | -1152.65285 | 11.13 |
| **PMe2/OTs** | | | | | | | | |
| TAu_E_1+_Ph_OTs | C23H28AuNO3PS | -2045.74433 | 0.50021 | 0.09788 | -2047.49571 | -2047.06107 | -1152.68755 | 0.00 |
| inherit F | C18H25AuNP | -1230.41292 | 0.41427 | 0.07494 | -1231.46896 | -1231.10491 | -1152.63524 | 32.82 |
| inherit G | C18H25AuNP | -1230.38523 | 0.41214 | 0.07306 | -1231.44037 | -1231.07718 | -1152.60751 | 50.23 |
| inherit H | C18H25AuNP | -1230.44501 | 0.41554 | 0.07360 | -1231.49609 | -1231.12986 | -1152.66019 | 17.17 |
| TAu_I_1+_CH2CH2Ph_OTs | C25H32AuNO3PS | -2124.19909 | 0.56080 | 0.10160 | -2126.04705 | -2125.55432 | -1152.71114 | -14.80 |

| Name | Formula | E SV(P) | Hcorr | TScorr | E TZVP | G | on scale | Grel |
|---|---|---|---|---|---|---|---|---|
| TAu_J_1+_Ph_EthOTs_extTS | C25H32AuNO3PS | -2124.11710 | 0.55796 | 0.10164 | -2125.97930 | -2125.48943 | -1152.64625 | 25.92 |
| TAu_K_1+_Ph_CH2CH2OTs | C25H32AuNO3PS | -2124.16867 | 0.55955 | 0.10204 | -2126.01335 | -2125.52216 | -1152.67898 | 5.38 |
| TAu_L_1+_Ph_CH2CH2OTs_rdelTS | C25H32AuNO3PS | -2124.13507 | 0.55798 | 0.09876 | -2125.97453 | -2125.48272 | -1152.63954 | 30.13 |
| TAu_Z_1+_Ph_Eth__OTs | C25H32AuNO3PS | -2124.12036 | 0.55817 | 0.10504 | -2125.98353 | -2125.49574 | -1152.65256 | 21.96 |
| **PAd2** | | | | | | | | |
| MAu_E_1+_Ph_OTf | C35H45AuF3NO3PS | -2811.38084 | 0.79925 | 0.11241 | -2814.03701 | -2813.31307 | -1852.05773 | 0.00 |
| MAu_F_2+_Ph_Eth | C36H49AuNP | -1929.37131 | 0.81904 | 0.09806 | -1931.24396 | -1930.49061 | -1852.02095 | 23.08 |
| MAu_G_2+_Ph_Eth_insTS | C36H49AuNP | -1929.34903 | 0.81754 | 0.09557 | -1931.22038 | -1930.46687 | -1851.99720 | 37.98 |
| MAu_H_2+_CH2CH2Ph_ortho | C36H49AuNP | -1929.41320 | 0.82135 | 0.09529 | -1931.28053 | -1930.52302 | -1852.05336 | 2.74 |
| MAu_I_1+_CH2CH2Ph_OTf | C37H49AuF3NO3PS | -2889.84191 | 0.85890 | 0.12003 | -2892.59523 | -2891.81676 | -1852.09175 | -21.35 |
| MAu_J_1+_Ph_EthOTf_extTS | C37H49AuF3NO3PS | -2889.76382 | 0.85586 | 0.11992 | -2892.52910 | -2891.75359 | -1852.02858 | 18.29 |
| MAu_K_1+_Ph_CH2CH2OTf_rot1 | C37H49AuF3NO3PS | -2889.80074 | 0.85833 | 0.11824 | -2892.55048 | -2891.77137 | -1852.04636 | 7.13 |
| MAu_L_1+_Ph_CH2CH2OTf_rdelTS_rot4 | C37H49AuF3NO3PS | -2889.76906 | 0.85542 | 0.11963 | -2892.51313 | -2891.73787 | -1852.01286 | 28.15 |
| MAu_Z_1+_Ph_Eth__OTf | C37H49AuF3NO3PS | -2889.76592 | 0.85737 | 0.12123 | -2892.53478 | -2891.75863 | -1852.03362 | 15.13 |
| **PAd2/Btn** | | | | | | | | |
| MAu_E_1+_Ph_OTf | C35H45AuF3NO3PS | -2811.38084 | 0.79925 | 0.11241 | -2814.03701 | -2813.31307 | -1852.05773 | 0.00 |
| MAu_F_2+_Ph_Btna | C38H53AuNP | -2007.83114 | 0.87919 | 0.10394 | -2009.79919 | -2008.98964 | -1852.01958 | 23.94 |
| MAu_F_2+_Ph_Btnb | C38H53AuNP | -2007.82877 | 0.87898 | 0.10310 | -2009.79626 | -2008.98636 | -1852.01630 | 26.00 |
| MAu_G_2+_Ph_Btn_12insTS | C38H53AuNP | -2007.81248 | 0.87637 | 0.10209 | -2009.77978 | -2008.97181 | -1852.00175 | 35.13 |
| MAu_G_2+_Ph_Btn_21insTS | C38H53AuNP | -2007.80399 | 0.87727 | 0.10251 | -2009.77067 | -2008.96209 | -1851.99203 | 41.23 |
| MAu_H_2+_CH2CHEtPh_ortho_X | C38H53AuNP | -2007.86872 | 0.87959 | 0.10284 | -2009.83266 | -2009.02196 | -1852.05190 | 3.65 |
| MAu_H_2+_CHEtCH2Ph_ortho_y | C38H53AuNP | -2007.86492 | 0.87917 | 0.10213 | -2009.82807 | -2009.01733 | -1852.04727 | 6.56 |
| MAu_I_1+_CH2CHEtPh_OTf_Y | C39H53AuF3NO3PS | -2968.29764 | 0.91781 | 0.12445 | -2971.14531 | -2970.31088 | -1852.08548 | -17.41 |
| MAu_I_1+_CHEtCH2Ph_OTf_X | C39H53AuF3NO3PS | -2968.29430 | 0.91808 | 0.12394 | -2971.14045 | -2970.30541 | -1852.08001 | -13.98 |
| MAu_J_1+_Ph_BtnOTf_12extTS | C39H53AuF3NO3PS | -2968.22990 | 0.91466 | 0.12443 | -2971.08926 | -2970.25797 | -1852.03257 | 15.79 |
| MAu_J_1+_Ph_BtnOTf_21extTS | C39H53AuF3NO3PS | -2968.22106 | 0.91557 | 0.12416 | -2971.07850 | -2970.24612 | -1852.02072 | 23.22 |
| MAu_K_1+_Ph_CH2CHEtOTf_X | C39H53AuF3NO3PS | -2968.26129 | 0.91764 | 0.12334 | -2971.10526 | -2970.27025 | -1852.04485 | 8.08 |
| MAu_K_1+_Ph_CHEtCH2OTf_Y | C39H53AuF3NO3PS | -2968.25151 | 0.91754 | 0.12409 | -2971.09549 | -2970.26110 | -1852.03569 | 13.83 |
| MAu_L_1+_Ph_CH2CHEtOTf_rdelTS_Y | C39H53AuF3NO3PS | -2968.22836 | 0.91475 | 0.12442 | -2971.06817 | -2970.23678 | -1852.01138 | 29.09 |

| *Name* | *Formula* | *E SV(P)* | *Hcorr* | *TScorr* | *E TZVP* | *G* | *on scale* | *Grel* |
|---|---|---|---|---|---|---|---|---|
| MAu_L_1+_Ph_CHEtCH2OTf_rdelTS_Y | C39H53AuF3NO3PS | -2968.21941 | 0.91521 | 0.12535 | -2971.05995 | -2970.22872 | -1852.00331 | 34.14 |
| MAu_Z_1+_Ph_Btn__OTf | C39H53AuF3NO3PS | -2968.23057 | 0.91613 | 0.12535 | -2971.09290 | -2970.26075 | -1852.03535 | 14.04 |
| **PAd2/Ebn** | | | | | | | | |
| MAu_E_1+_Ph_OTf | C35H45AuF3NO3PS | -2811.38084 | 0.79925 | 0.11241 | -2814.03701 | -2813.31307 | -1852.05773 | 0.00 |
| MAu_F_2+_Ph_Ebn | C40H57AuNP | -2086.28690 | 0.93751 | 0.10822 | -2088.34936 | -2087.48436 | -1852.01664 | 25.78 |
| MAu_G_2+_Ph_Ebn_12insTS | C40H57AuNP | -2086.27280 | 0.93483 | 0.10869 | -2088.33528 | -2087.47327 | -1852.00556 | 32.74 |
| MAu_G_2+_Ph_Ebn_21insTS | C40H57AuNP | -2086.24527 | 0.93701 | 0.10691 | -2088.30683 | -2087.44145 | -1851.97374 | 52.70 |
| MAu_H_2+_CEt2CH2Ph_ortho_X | C40H57AuNP | -2086.29961 | 0.93853 | 0.10674 | -2088.35707 | -2087.49006 | -1852.02234 | 22.20 |
| MAu_H_2+_CH2CEt2Ph_ortho_X | C40H57AuNP | -2086.32240 | 0.93822 | 0.10792 | -2088.38287 | -2087.51696 | -1852.04925 | 5.32 |
| MAu_I_1+_CEt2CH2Ph_OTf_X | C41H57AuF3NO3PS | -3046.70507 | 0.97762 | 0.12919 | -3049.64489 | -3048.75383 | -1852.03077 | 16.92 |
| MAu_I_1+_CH2CEt2Ph_OTf_X | C41H57AuF3NO3PS | -3046.73361 | 0.97744 | 0.12832 | -3049.67719 | -3048.78572 | -1852.06266 | -3.10 |
| MAu_J_1+_Ph_EbnOTf_12extTS | C41H57AuF3NO3PS | -3046.68904 | 0.97382 | 0.12875 | -3049.64137 | -3048.75381 | -1852.03076 | 16.92 |
| MAu_J_1+_Ph_EbnOTf_21extTS | C41H57AuF3NO3PS | -3046.64882 | 0.97512 | 0.12951 | -3049.59877 | -3048.71043 | -1851.98737 | 44.15 |
| MAu_K_1+_Ph_CEt2CH2OTf | C41H57AuF3NO3PS | -3046.67666 | 0.97734 | 0.12846 | -3049.61534 | -3048.72406 | -1852.00101 | 35.59 |
| MAu_K_1+_Ph_CH2CEt2OTf | C41H57AuF3NO3PS | -3046.70762 | 0.97608 | 0.12744 | -3049.64429 | -3048.75359 | -1852.03053 | 17.07 |
| MAu_L_1+_Ph_CEt2CH2OTf_rdelTS_X | C41H57AuF3NO3PS | -3046.64842 | 0.97454 | 0.12790 | -3049.58566 | -3048.69681 | -1851.97375 | 52.70 |
| MAu_L_1+_Ph_CH2CEt2OTf_rdelTS_X | C41H57AuF3NO3PS | -3046.67928 | 0.97379 | 0.12734 | -3049.61449 | -3048.72602 | -1852.00296 | 34.37 |
| MAu_Z_1+_Ph_Ebn__OTf | C41H57AuF3NO3PS | -3046.69036 | 0.97499 | 0.13330 | -3049.64695 | -3048.76127 | -1852.03822 | 12.24 |
| **PAd2/OTs** | | | | | | | | |
| MTAu_E_1+_Ph_OTs | C41H52AuNO3PS | -2744.70785 | 0.90498 | 0.11932 | -2747.27436 | -2746.44932 | -1852.07580 | 0.00 |
| inherit F | C36H49AuNP | -1929.34903 | 0.81754 | 0.09806 | -1931.24396 | -1930.49061 | -1852.02095 | 34.42 |
| inherit G | C36H49AuNP | -1929.41320 | 0.82135 | 0.09557 | -1931.22038 | -1930.46687 | -1851.99720 | 49.32 |
| inherit H | C37H49AuF3NO3PS | -2889.84191 | 0.85890 | 0.09529 | -1931.28053 | -1930.52302 | -1852.05336 | 14.08 |
| MTAu_I_1+_CH2CH2Ph_OTs | C43H56AuNO3PS | -2823.16602 | 0.96501 | 0.12575 | -2825.82985 | -2824.94909 | -1852.10591 | -18.89 |
| MTAu_J_1+_Ph_CH2CH2OTs_extTS | C43H56AuNO3PS | -2823.07843 | 0.96189 | 0.12322 | -2825.75715 | -2824.87781 | -1852.03463 | 25.83 |
| MTAu_K_1+_Ph_CH2CH2OTs | C43H56AuNO3PS | -2823.13100 | 0.96462 | 0.12453 | -2825.79201 | -2824.91082 | -1852.06764 | 5.12 |
| MTAu_L_1+_Ph_CH2CH2OTs_rdelTS | C43H56AuNO3PS | -2823.10268 | 0.96154 | 0.12428 | -2825.75882 | -2824.88055 | -1852.03737 | 24.12 |
| MTAu_Z_1+_Ph_CH2CH2__OTs | C43H56AuNO3PS | -2823.07903 | 0.96315 | 0.12545 | -2825.75737 | -2824.87827 | -1852.03509 | 25.55 |
| **anisyl** | | | | | | | | |

| **Name** | **Formula** | **E SV(P)** | **Hcorr** | **TScorr** | **E TZVP** | **G** | **on scale** | **Grel** |
|---|---|---|---|---|---|---|---|---|
| AAu_E_1+_Ph_OTf | C18H23AuF3NO4PS | -2226.72657 | 0.42931 | 0.09608 | -2228.70634 | -2228.34141 | -1267.08606 | 0.00 |
| AAu_F_2+_Ph_Eth | C19H27AuNOP | -1344.72214 | 0.45030 | 0.07933 | -1345.91660 | -1345.51944 | -1267.04978 | 22.77 |
| AAu_G_2+_Ph_Eth_insTS | C19H27AuNOP | -1344.69922 | 0.44773 | 0.07897 | -1345.89283 | -1345.49800 | -1267.02834 | 36.22 |
| AAu_H_2+_CH2CH2Ph_ortho | C19H27AuNOP | -1344.75655 | 0.45092 | 0.07952 | -1345.94557 | -1345.54794 | -1267.07827 | 4.89 |
| AAu_I_1+_CH2CH2Ph_OTf | C20H27AuF3NO4PS | -2305.18280 | 0.48972 | 0.10151 | -2307.25882 | -2306.83711 | -1267.11211 | -16.34 |
| AAu_J_1+_Ph_EthOTf_extTS | C20H27AuF3NO4PS | -2305.11159 | 0.48670 | 0.10271 | -2307.19917 | -2306.78128 | -1267.05627 | 18.69 |
| AAu_K_1+_Ph_CH2CH2OTf | C20H27AuF3NO4PS | -2305.14270 | 0.48868 | 0.10159 | -2307.21535 | -2306.79473 | -1267.06972 | 10.25 |
| AAu_L_1+_Ph_CH2CH2OTf_rdelTS | C20H27AuF3NO4PS | -2305.10795 | 0.48702 | 0.10060 | -2307.17367 | -2306.75405 | -1267.02904 | 35.78 |
| AAu_Z_1+_Ph_Eth__OTf | C20H27AuF3NO4PS | -2305.11715 | 0.48717 | 0.10550 | -2307.20757 | -2306.79109 | -1267.06608 | 12.54 |
| **C6F4** | | | | | | | | |
| FAu_E_1+_Ph_OTf | C17H17AuF7NO3PS | -2508.72009 | 0.36578 | 0.09685 | -2511.03472 | -2510.73383 | -1549.47849 | 0.00 |
| FAu_F_2+_Ph_Eth | C18H21AuF4NP | -1626.71272 | 0.38534 | 0.08450 | -1628.24217 | -1627.91344 | -1549.44377 | 21.78 |
| FAu_G_2+_Ph_Eth_insTS | C18H21AuF4NP | -1626.68739 | 0.38354 | 0.08063 | -1628.21593 | -1627.88640 | -1549.41674 | 38.75 |
| FAu_H_2+_CH2CH2Ph_ortho | C18H21AuF4NP | -1626.74617 | 0.38681 | 0.08147 | -1628.27102 | -1627.93879 | -1549.46912 | 5.88 |
| FAu_I_1+_CH2CH2Ph_OTf | C19H21AuF7NO3PS | -2587.17701 | 0.42591 | 0.10428 | -2589.58817 | -2589.23213 | -1549.50712 | -17.96 |
| FAu_J_1+_Ph_EthOTf_extTS | C19H21AuF7NO3PS | -2587.10516 | 0.42263 | 0.10334 | -2589.52776 | -2589.17437 | -1549.44936 | 18.28 |
| FAu_K_1+_Ph_CH2CH2OTf | C19H21AuF7NO3PS | -2587.14036 | 0.42476 | 0.10460 | -2589.54762 | -2589.19295 | -1549.46794 | 6.62 |
| FAu_L_1+_Ph_CH2CH2OTf_rdelTS | C19H21AuF7NO3PS | -2587.10707 | 0.42287 | 0.10392 | -2589.50843 | -2589.15518 | -1549.43017 | 30.32 |
| FAu_Z_1+_Ph_Eth__OTf | C19H21AuF7NO3PS | -2587.10981 | 0.42328 | 0.10673 | -2589.53478 | -2589.18301 | -1549.45800 | 12.86 |
| **PMe3/NMe3** | | | | | | | | |
| OAu_E_1+_Ph_OTf | C13H23AuF3NO3PS | -1961.48278 | 0.38991 | 0.08556 | -1963.16188 | -1962.82929 | -1001.57395 | 0.00 |
| OAu_F_2+_Ph_Eth | C14H27AuNP | -1079.47724 | 0.40971 | 0.07201 | -1080.37266 | -1080.01120 | -1001.54153 | 20.34 |
| OAu_G_2+_Ph_Eth_insTS | C14H27AuNP | -1079.45555 | 0.40779 | 0.07071 | -1080.34914 | -1079.98874 | -1001.51907 | 34.44 |
| OAu_H_2+_CH2CH2Ph_ortho | C14H27AuNP | -1079.50833 | 0.41130 | 0.07078 | -1080.39779 | -1080.03391 | -1001.56425 | 6.09 |
| OAu_I_1+_CH2CH2Ph_OTf | C15H27AuF3NO3PS | -2039.93767 | 0.44997 | 0.09505 | -2041.71240 | -2041.32611 | -1001.60110 | -17.04 |
| OAu_J_1+_Ph_EthOTf_extTS | C15H27AuF3NO3PS | -2039.86852 | 0.44737 | 0.09194 | -2041.65625 | -2041.27048 | -1001.54548 | 17.87 |
| OAu_K_1+_Ph_CH2CH2OTf | C15H27AuF3NO3PS | -2039.90254 | 0.44916 | 0.09342 | -2041.67415 | -2041.28758 | -1001.56257 | 7.14 |
| OAu_L_1+_Ph_CH2CH2OTf_rdelTS | C15H27AuF3NO3PS | -2039.86620 | 0.44641 | 0.09347 | -2041.63146 | -2041.24767 | -1001.52266 | 32.18 |
| OAu_Z_1+_Ph_Eth__OTf | C15H27AuF3NO3PS | -2039.87125 | 0.44796 | 0.09483 | -2041.66368 | -2041.27925 | -1001.55424 | 12.37 |

| ***Name*** | ***Formula*** | ***E SV(P)*** | ***Hcorr*** | ***TScorr*** | ***E TZVP*** | ***G*** | ***on scale*** | ***Grel*** |
|---|---|---|---|---|---|---|---|---|
| **PMe2/PMe2** | | | | | | | | |
| PAu_E_1+_Ph_OTf | C17H21AuF3O3P2S | -2398.90949 | 0.38831 | 0.09320 | -2400.83376 | -2400.50788 | -1439.25254 | 0.00 |
| PAu_F_2+_Ph_Eth | C18H25AuP2 | -1516.91087 | 0.40841 | 0.07705 | -1518.04994 | -1517.69316 | -1439.22349 | 18.23 |
| PAu_G_2+_Ph_Eth_insTS | C18H25AuP2 | -1516.89511 | 0.40629 | 0.07604 | -1518.03279 | -1517.67745 | -1439.20778 | 28.09 |
| PAu_H_2+_CH2CH2Ph_ortho | C18H25AuP2 | -1516.94296 | 0.40921 | 0.07657 | -1518.07704 | -1517.71913 | -1439.24947 | 1.93 |
| PAu_I_1+_CH2CH2Ph_OTf | C19H25AuF3O3P2S | -2477.36340 | 0.44808 | 0.10086 | -2479.38521 | -2479.00471 | -1439.27970 | -17.04 |
| PAu_J_1+_Ph_EthOTf_extTS | C19H25AuF3O3P2S | -2477.30295 | 0.44484 | 0.10025 | -2479.33364 | -2478.95597 | -1439.23096 | 13.54 |
| PAu_K_1+_Ph_CH2CH2OTf | C19H25AuF3O3P2S | -2477.33683 | 0.44752 | 0.09754 | -2479.35058 | -2478.96842 | -1439.24341 | 5.73 |
| PAu_L_1+_Ph_CH2CH2OTf_rdelTS | C19H25AuF3O3P2S | -2477.27920 | 0.44543 | 0.09782 | -2479.29014 | -2478.91025 | -1439.18524 | 42.23 |
| PAu_Z_1+_Ph_Eth__OTf | C19H25AuF3O3P2S | -2477.30957 | 0.44560 | 0.10273 | -2479.34381 | -2478.96703 | -1439.24202 | 6.60 |
| **PH2** | | | | | | | | |
| YAu_E_1+_Ph_OTf | C15H17AuF3NO3PS | -2033.92528 | 0.33355 | 0.08331 | -2035.66705 | -2035.38932 | -1074.13397 | 0.00 |
| YAu_F_2+_Ph_Eth | C16H21AuNP | -1151.92106 | 0.35365 | 0.06839 | -1152.87762 | -1152.56980 | -1074.10013 | 21.24 |
| YAu_G_2+_Ph_Eth_insTS | C16H21AuNP | -1151.89765 | 0.35199 | 0.06679 | -1152.85296 | -1152.54572 | -1074.07606 | 36.34 |
| YAu_H_2+_CH2CH2Ph_ortho | C16H21AuNP | -1151.95338 | 0.35500 | 0.06730 | -1152.90495 | -1152.59505 | -1074.12538 | 5.39 |
| YAu_I_1+_CH2CH2Ph_OTf | C17H21AuF3NO3PS | -2112.38344 | 0.39368 | 0.08975 | -2114.22079 | -2113.88724 | -1074.16223 | -17.73 |
| YAu_J_1+_EthOTf_extTS | C17H21AuF3NO3PS | -2112.31420 | 0.39124 | 0.08824 | -2114.16418 | -2113.83206 | -1074.10705 | 16.89 |
| YAu_K_1+_Ph_CH2CH2OTf | C17H21AuF3NO3PS | -2112.35116 | 0.39349 | 0.08749 | -2114.18471 | -2113.84984 | -1074.12483 | 5.74 |
| YAu_L_1+_Ph_CH2CH2OTf_rdelTS | C17H21AuF3NO3PS | -2112.31528 | 0.39120 | 0.08994 | -2114.14494 | -2113.81400 | -1074.08899 | 28.23 |
| YAu_Z_1+_Ph_Eth__OTf | C17H21AuF3NO3PS | -2112.31620 | 0.39150 | 0.09187 | -2114.16937 | -2113.83942 | -1074.11441 | 12.27 |

*Table S2. Relative free energies (kcal/mol).*

| entry | | L[a] | K | J[a] | Z | E | F | G | H[a] | I |
|---|---|---|---|---|---|---|---|---|---|---|
| 1 | PMe2 | 35.48 | 9.97 | 19.20 | 13.12 | 0.00 | 22.18 | 39.58 | 6.53 | -16.40 |
| 2 | PMe2/Btn | 36.33 | 16.10 | 17.16 | 12.70 | 0.00 | 21.25 | 38.52 | 8.12 | -14.28 |
| | | *43.77* | | *22.70* | | | | *43.19* | | |
| 3 | PMe2/Ebn | 44.98 | 17.74 | 16.40 | 11.13 | 0.00 | 23.43 | 37.86 | 10.51 | -7.18 |
| | | *52.50* | | *21.13* | | | | *48.87* | | |
| 4 | PMe2/OTs | 30.13 | 5.38 | 25.92 | 21.96 | 0.00 | 32.82 | 50.23 | 17.17 | -14.80 |
| 5 | PAd2 | 28.15 | 7.13 | 18.29 | 15.13 | 0.00 | 23.08 | 37.98 | 2.74 | -21.35 |
| 6 | PAd2/Btn | 29.09 | 8.08 | 15.79 | 14.04 | 0.00 | 23.94 | 35.13 | 3.65 | -17.41 |
| | | *34.14* | | *23.22* | | | | *41.23* | | |
| 7 | PAd2/Ebn | 34.37 | 17.07 | 16.92 | 12.24 | 0.00 | 25.78 | 32.74 | 5.32 | -3.10 |
| | | *52.70* | | *44.15* | | | | *52.70* | | |
| 8 | PAd2/OTs | 24.12 | 5.12 | 25.83 | 25.55 | 0.00 | 34.42 | 49.32 | 14.08 | -18.89 |
| 9 | anisyl | 35.78 | 10.25 | 18.69 | 12.54 | 0.00 | 22.77 | 36.22 | 4.89 | -16.34 |
| 10 | C6F4 | 30.32 | 6.62 | 18.28 | 12.86 | 0.00 | 21.78 | 38.75 | 5.88 | -17.96 |
| 11 | PMe3/NMe3 | 32.18 | 7.14 | 17.87 | 12.37 | 0.00 | 20.34 | 34.44 | 6.09 | -17.04 |
| 12 | PMe2/PMe2 | 42.23 | 5.73 | 13.54 | 6.60 | 0.00 | 18.23 | 28.09 | 1.93 | -17.04 |
| 13 | PH2 | 28.23 | 5.74 | 16.89 | 12.27 | 0.00 | 21.24 | 36.34 | 36.34 | -17.73 |

[a] for species **G**, **J** and **L** 2,1 values in italics.
